# Supplementary material for: Deep learning image analysis for continuous single-cell imaging of dynamic processes in Plasmodium falciparum-infected erythrocytes
Source: Commun Biol. 2025 Mar 25;8:487. doi: 10.1038/s42003-025-07894-3 (PMC11937545; doi:10.1038/s42003-025-07894-3)
Supplement: Supplementary file 1 — Supplementary Information [file 42003_2025_7894_MOESM1_ESM.docx]

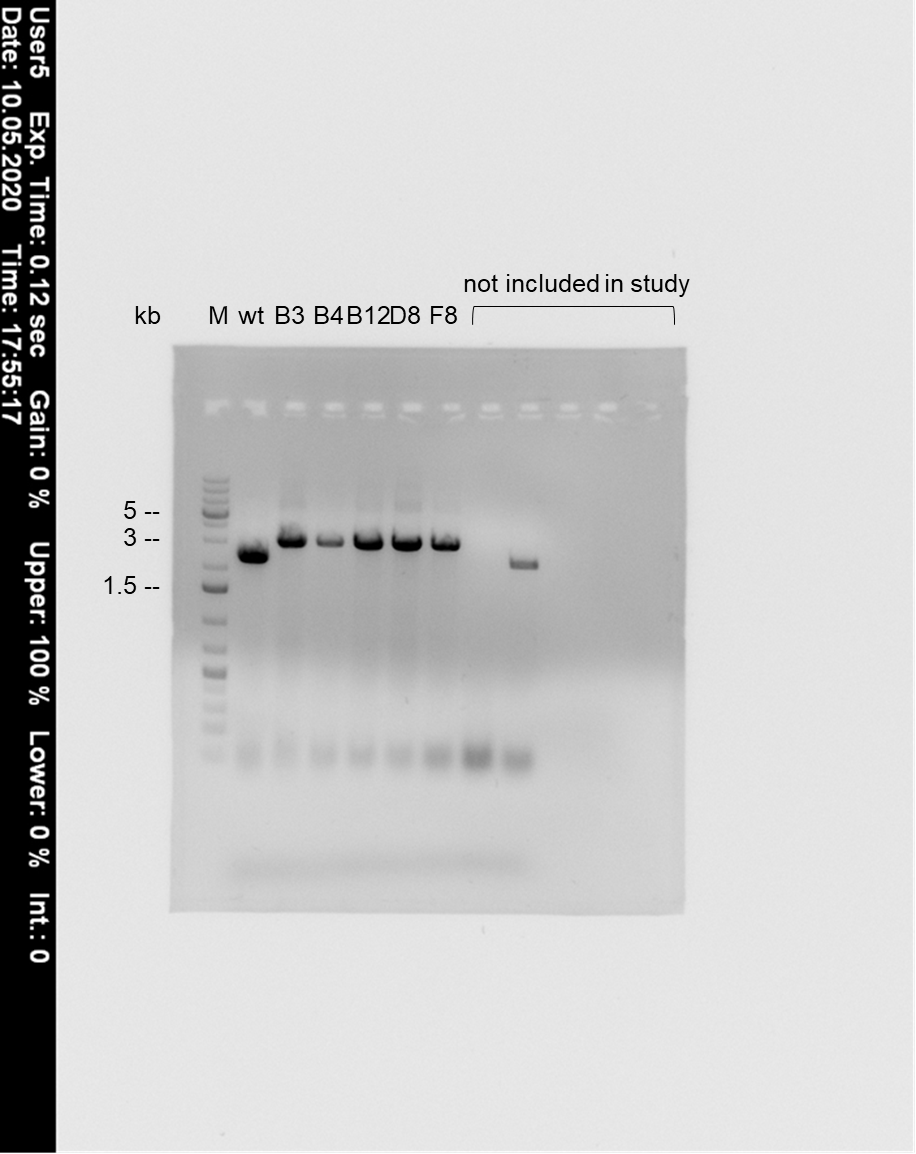


**Supplementary Figure 1.** Uncropped image of Figure 4B. Marker: Thermo Scientific generuler 1 kb Plus DNA ladder.


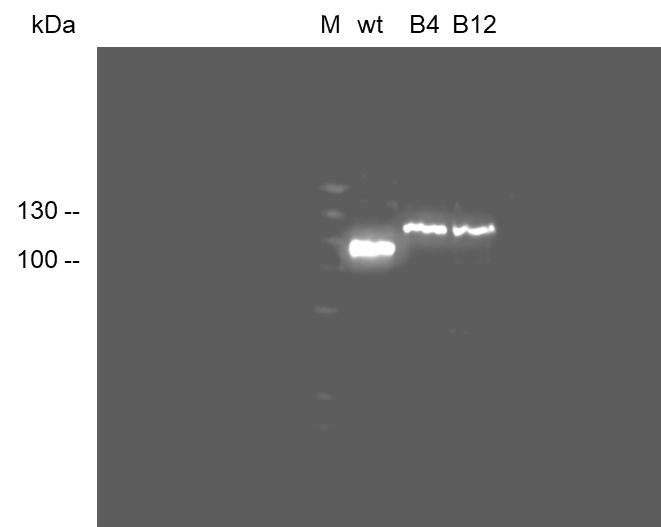


**Supplementary Figure 2.** Uncropped image of Figure 4C. Marker: Thermo Scientific PageRuler Plus prestained, 10-250 kDa,.
