# Supplementary material for: Deep learning image analysis for continuous single-cell imaging of dynamic processes in Plasmodium falciparum-infected erythrocytes
Source: Commun Biol. 2025 Mar 25;8:487. doi: 10.1038/s42003-025-07894-3 (PMC11937545; doi:10.1038/s42003-025-07894-3)
Supplement: Supplementary file 2 — Description of Additional Supplementary Files [file 42003_2025_7894_MOESM2_ESM.docx]

**Description of Additional Supplementary Files**

File name: Supplementary Data
Description: The file contains the source data underpinning Figure 4E, Figure 4F, Figure 4G, Figure 6A, Figure 6B, Figure 7B, and Figure 7C.
